# Preparation of the nursing workforce in the field of women’s health

**DOI:** 10.4069/whn.2024.05.30

**Published:** 2024-06-28

**Authors:** Sukhee Ahn

**Affiliations:** College of Nursing, Chungnam National University, Seoul, Korea

## Introduction

In hospitals where deliveries take place, there is a critical shortage of obstetricians, leading to extended waiting times and a preference for cesarean sections over vaginal deliveries, which require more unpredictable and longer periods of labor and may not be as amenable to scheduling as elective cesarean sections. This shortage is a significant factor behind the rise in the cesarean section rate, which reached 58.7% in 2021 [1]. Despite efforts since 2011 to support facilities in regions lacking obstetric care through funding for infrastructure, equipment, and operational costs, new underserved areas continue to emerge as obstetric clinics close and services are discontinued [2]. This situation underscores the need for measures to protect the health rights of women of childbearing age. Additionally, with fewer candidates applying to obstetrics and gynecology residency programs, hospitals increasingly rely on obstetrics clinical nurses to assist obstetricians, alongside nurses specializing in women’s health care [3].

In 2023, South Korea’s total fertility rate was recorded at 0.72—less than half of the OECD average of 1.58 in 2021 [4]. In response, the government, the National Assembly, and local governments have initiated various policy projects to address the low birth rates, leading to a heightened demand for nursing personnel in women’s health to support pregnant women and their families [5]. Additionally, from 2020 to 2023, the coronavirus disease 2019 (COVID-19) pandemic increased stress and fear among pregnant women, particularly concerning the risk of COVID-19 infection during delivery, influencing their choice of delivery method [6]. The critical role of nursing personnel in supporting pregnant women and their families during such crises has been increasingly recognized. Therefore, this editorial aims to propose strategies for preparing nursing workforce to address the evolving needs in women’s health care, including the increase in underserved areas for deliveries, the heightened health needs of pregnant women during infectious disease outbreaks, and various maternal health and childcare support projects designed to combat low birth rates.

## Current status of nursing workforce preparation in the field of women’s health

Under the Medical Service Act in South Korea, a midwife is defined as either a person who holds a nursing license and has completed a 1-year midwifery internship at a medical institution approved by the Minister of Health and Welfare, or someone who has obtained a foreign midwifery license recognized by the same authority and has passed the national examination [7]. According to the guidelines for field midwifery practice, midwives are responsible for performing 56 tasks across seven areas: pregnancy management, delivery management, postpartum care, newborn care, primary healthcare, law and ethics, and general management [8]. However, due to a declining birth rate and reduced hiring of midwives in hospitals, the number of midwifery internship institutions has dwindled to four as of 2024, training only 12 midwives this year [5]. Additionally, Article 38 of the Medical Service Act Enforcement Rule mandates that general hospitals—and hospitals or clinics with obstetrics departments—must staff at least one-third of their delivery room nurses as midwives. Despite this requirement, compliance is low due to insufficient regulations [5].

Since the maternal fetal intensive care unit (MFICU) was established in 2014 to efficiently manage high-risk pregnant mothers and newborns, there has been a recognized need to ensure that MFICU nurses possess the requisite expertise to care for this vulnerable population. In response, a collaborative effort involving the Korean Association of Women Health Nurses, the Korean Society of Women Health Nursing, and the Korean Society of Maternal and Child Health led to the development and evaluation of an empowering education program aimed at enhancing the practical competencies of MFICU nurses [9]. This education is provided to professionals in the field every year. Despite these efforts, there remains a lack of a specific training plan for MFICU nurses that is led by either the government or medical professionals.

## Performance of the nursing workforce in the field of women’s health

The performance of the nursing workforce in women’s health provides crucial evidence for the future allocation of necessary personnel. One study introduced a nurse-led doula support program as a natural childbirth method and assessed its effectiveness. The findings indicated that this program decreased maternal anxiety and labor pain while increasing childbirth satisfaction [10]. Another study examined the perinatal health outcomes for mothers who chose midwife-led natural childbirth at birthing centers. It found that these outcomes were superior to those of mothers who delivered in hospitals [11]. Specifically, mothers at birthing centers experienced lower risks of perineal tears, postpartum hemorrhage, and cesarean sections, along with fewer complications related to newborn Apgar scores and meconium staining. These findings suggest that women who actively choose and engage in natural childbirth, supported by professionally skilled midwives providing personalized labor and delivery care, can achieve better health outcomes and a more positive childbirth experience for themselves and their families. This highlights the importance of maintaining staffing standards for midwives in hospitals and ensuring an adequate number of nurses with specialized education in perinatal care to improve perinatal health outcomes and ensure a safe and joyful childbirth experience.

To address the issue of a low birth rate, the government has revised sections of the Mother and Child Health Act (Articles 10 and 11). These amendments assign the responsibility of home visits to national and local governments to support pregnant women, infants, premature babies, and others. These visits are conducted by maternal and child health professionals, including midwives, to provide essential health management services [12]. In recent efforts to respond to the low birth rate, professionals specializing in women’s health nursing have joined as key contributors to various initiatives. The Korean Midwives Association has collaborated with the “Seoul Breastfeeding Support Project,” which has been operational since July 2023. This partnership focuses on training the required nursing staff and has documented the accomplishments of those deployed in Seoul [13]. The Korean Midwives Association offers an 8-week course, totaling 48 hours, to train breastfeeding specialists [14]. The Seoul project team then certifies these individuals as Seoul Breastfeeding Counselors, who deliver personalized care and breastfeeding education to mothers on a one-to-one basis. Among the 5,400 mothers involved in this project, 98% who responded to a survey reported satisfaction with the services provided. Impressively, 71% of the mothers who utilized these services continued to practice breastfeeding (including mixed feeding) 3 months postpartum [13]. This figure significantly exceeds the national average, as only 26.9% of mothers reported breastfeeding at 3 months postpartum in 2021 [15]. This contrast demonstrates the significant contribution of well-prepared nursing personnel, such as breastfeeding counselors, to breastfeeding practices.

## Expansion of women’s health nursing providers’ roles to meet health needs

Based on the experience of the COVID-19 pandemic, nurses specializing in women’s health must prepare to take on proactive roles as educators and counselors. This is essential to address the physical and mental health needs of pregnant women in the face of future infectious disease outbreaks. While the role of midwives in medical institutions and birthing centers is crucial, there is a need to broaden their scope of practice. This expansion should include compliance with regulations that enable midwives to manage childbirth and maternal and newborn health in underserved areas, such as rural and fishing communities lacking obstetricians, thereby safeguarding women’s rights to childbirth.

A study exploring the scope of practice for midwives in countries such as Sweden, the United Kingdom, and the United States [16] found that the role of midwives extends beyond perinatal health management. Their responsibilities also encompass education on parenting and contraception/family planning for children and adolescents, as well as health education, counseling on domestic violence, substance abuse, mental illness, school maladjustment, and providing primary health care and health education to community residents. Given the health needs in South Korea, it is crucial to conduct research on expanding the scope of women’s health nursing practices and to engage in policy-making to broaden these roles. In the future, nurses specializing in women’s health should be equipped to perform roles that go beyond hospital-based perinatal health management, offering primary health care and mental health support throughout women’s life cycles.

## Proposals for improving the training of nursing personnel in the field of women’s health

To address the issue of low birth rates and adapt to the evolving healthcare landscape, the operation of the midwifery internship program should be improved and specialized competency education for nursing personnel in women’s health should be promoted. The Korean Midwives Association has urgently called for the training of well-prepared midwives to respond to low birth rates. They advocate for relaxing the standards for midwifery internship institutions to increase the number of these institutions and to include educational entities outside of medical facilities [5]. To remedy the deficiencies in the systematic operation and management of educational programs at midwifery internship medical institutions, it is suggested that education in midwifery be handled by specialized organizations such as university educational institutions or the Korean Midwives Association. Practical training should be conducted at designated internship medical institutions, emphasizing hands-on perinatal nursing practice and midwifery training to improve educational efficiency.

Another approach involves integrating midwifery into the graduate-level advanced practice nursing program. Currently, nurse-midwives who are trained through the midwifery internship process are recognized as medical personnel, yet they are not considered advanced practice nurses. In contrast, in the United States, midwives undergo training in graduate master’s programs that include advanced practice nursing education, actively engaging in specialized nursing practices in women’s health. Therefore, South Korea should establish a separate 2-year midwifery/women’s health advanced practice nursing program within graduate nursing schools. This program would run concurrently with the existing 1-year midwifery internship program, thereby broadening the training opportunities for specialized personnel in women’s health, including midwives. To implement these improvements, collaboration with the Ministry of Health and Welfare, the Korean Nursing Association, the Korean Midwives Association, and the Korean Accreditation Board of Nursing Education is needed.

To improve the competencies of women’s health nurses and obstetrics clinical nurses in obstetrics and gynecology departments, hospitals should implement systematic job competency education and training programs in collaboration with specialized organizations in women’s health. Additionally, these nurses should first have the opportunity to participate in midwifery internships, enabling them to acquire midwifery licenses and practice with greater expertise. This approach will ensure the provision of safe nursing care to pregnant women and other clients in women’s health, leading to better health outcomes.

## Conclusion

This study examined the training processes for nursing personnel specializing in women’s health, focusing on their role in managing the health of pregnant women and infants. This exploration occurred within the framework of policies designed to address the challenges of a changing healthcare landscape and declining birth rates. The study assessed the impact of expanded roles for these professionals and suggested strategies to enhance both their numbers and their capabilities. To effectively respond to these challenges, stakeholders in women’s health must work collaboratively across disciplines to develop and implement training programs. These programs should provide the essential skills needed for projects at both national and local levels. Such initiatives are expected to significantly boost the engagement of well-trained nursing personnel, thereby contributing to public health.
